# Supplementary material for: Timing of Entry into Paid Employment, Adverse Physical Work Exposures and Health: The Young Helsinki Health Study
Source: Int J Environ Res Public Health. 2020 Oct 27;17(21):7854. doi: 10.3390/ijerph17217854 (PMC7662500; doi:10.3390/ijerph17217854)
Supplement: Supplementary file 1 [file ijerph-17-07854-s001.pdf]

Supplementary Materials

Timing of Entry into Paid Employment, Adverse Physical Work Exposures and Health: The Young Helsinki Health Study

Tea Lallukka [1,2,*], Rahman Shiri [2], Olli Pietiläinen [1], Johanna Kausto [2], Hilla Sumanen [1,3], Jaana I. Halonen [4], Eero Lahelma [1], Ossi Rahkonen [1], Minna Mänty [1,5] and Anne Kouvonen [6,7]

[1] Department of Public Health, University of Helsinki, P.O. Box 20, 00014 Helsinki, Finland; olli.k.pietilainen@helsinki.fi (O.P.); hilla.sumanen@helsinki.fi (H.S.); eero.lahelma@helsinki.fi (E.L.); ossi.rahkonen@helsinki.fi (O.R.); minna.manty@helsinki.fi (M.M.)

[2] Finnish Institute of Occupational Health, P.O. Box 18, 00032 Helsinki, Finland; rahman.shiri@ttl.fi (R.S.); johanna.kausto@ttl.fi (J.K.)

[3] Department of Health Care and Emergency Care, South Eastern Finland University of Applied Sciences, 48220 Kotka, Finland

[4] Department of Health Security, Finnish Institute for Health and Welfare, P.O. Box 30, 00271 Helsinki, Finland; jaana.halonen@thl.fi

[5] Department of Strategy and Research, City of Vantaa, 01030 Vantaa, Finland

[6] Faculty of Social Sciences, 00014 University of Helsinki, Helsinki, Finland; anne.kouvonen@helsinki.fi

[7] Research Institute of Psychology, SWPS University of Social Sciences and Humanities, 53-238 Wroclaw, Poland

***** Correspondence: tea.lallukka@helsinki.fi; Tel.: +358-505-704-399

Received: 12 September 2020; Accepted: 23 October 2020; Published: date

[*] Correspondence: tea.lallukka@helsinki.fi; Tel.: +358 50 57 04 39 9

Table S1: Gender–specific age-adjusted odds ratios (OR) for the associations between workload factors and health outcomes

Workload factor	Women				Men			
	Poor self-rated health		Common mental disorders		Poor self-rated health		Common mental disorders	
	OR	95% CI	OR	95% CI	OR	95% CI	OR	95% CI
Age at first employment (ref: 22-24 years)								
≤18	1.29	0.97-1.72	1.13	0.92-1.38	0.98	0.54-1.78	0.86	0.54-1.38
19–21	1.27	0.99-1.63	1.29	1.08-1.53	1.19	0.72-1.96	0.97	0.64-1.45
≥25	0.79	0.60-1.04	1.15	0.96-1.38	0.73	0.43-1.25	1.00	0.67-1.51
Heavy physical exertion or lifting and carrying heavy loads	1.49	1.21-1.83	1.22	1.06-1.40	2.08	1.38-3.14	0.99	0.72-1.37
Uncomfortable working postures	1.81	1.46-2.25	1.45	1.27-1.66	2.33	1.55-3.48	1.10	0.84-1.46
Trunk rotation	1.62	1.33-1.98	1.19	1.05-1.36	2.03	1.36-3.02	1.01	0.75-1.36
Repetitive movements	1.77	1.45-2.17	1.40	1.23-1.60	2.11	1.42-3.15	1.27	0.95-1.71
Sitting	1.34	1.09-1.64	1.18	1.03-1.34	1.93	1.30-2.88	2.07	1.56-2.75
Standing	2.20	1.75-2.78	1.80	1.52-2.13	2.47	1.55-3.94	2.11	1.46-3.05
Vibration	0.96	0.52-1.77	1.18	0.81-1.73	1.88	1.14-3.10	1.09	0.73-1.65

Table S2: Gender–specific age-adjusted odds ratios (OR) for the associations between behavior-related risk factors and health outcomes

Behavior-related risk factor	Women				Men			
	Poor self-rated health		Common mental disorders		Poor self-rated health		Common mental disorders	
	OR	95% CI	OR	95% CI	OR	95% CI	OR	95% CI
Smoking (ref: never)								
Past	1.40	1.10-1.79	1.27	1.08-1.50	1.54	0.98-2.41	1.26	0.89-1.79
Occasional	1.37	1.01-1.85	1.21	0.98-1.48	1.20	0.69-2.08	1.52	1.03-2.26
Current	2.27	1.75-2.96	1.58	1.29-1.94	3.25	1.97-5.36	1.68	1.07-2.66
Body mass index (ref: healthy weight)								
Underweight	1.68	0.86-3.29	1.13	0.73-1.74	1.82	0.22-14.90	0.28	0.04-2.23
Overweight	2.24	1.77-2.84	1.08	0.93-1.27	1.93	1.25-2.99	0.82	0.60-1.11
Obesity	4.95	3.90-6.27	1.37	1.14-1.65	5.37	3.32-8.68	0.98	0.65-1.49
Leisure-time physical activity (tertile, ref: low)								
Moderate	0.49	0.39-0.63	1.00	0.86-1.17	0.43	0.27-0.70	0.64	0.45-0.91
High	0.38	0.29-0.49	0.89	0.76-1.05	0.29	0.18-0.47	0.63	0.45-0.88
Binge drinking (ref: never)								
Less than once a month	1.04	0.82-1.33	0.99	0.85-1.15	0.51	0.30-0.89	1.00	0.64-1.57
Once a month	1.38	1.00-1.90	1.52	1.24-1.86	0.57	0.32-1.02	1.23	0.77-1.96
At least once a week	2.91	1.86-4.48	1.63	1.16-2.29	0.66	0.35-1.27	1.72	1.04-2.85

Table S3. Age- and gender-adjusted and multivariable odds ratios (OR) for the associations between age at first employment, gender, behavior-related risk factors, workload factors and low back pain

Characteristic	Age- and gender-adjusted model		Multivariable model	
	OR	95% CI	OR[1]	95% CI
Age at first employment[2] (ref: 22–24 years)				
≤18	1.16	0.94–1.44		
19–21	1.12	0.93–1.35		
≥25	0.89	0.73–1.08		
Gender, men vs. women	0.77	0.65-0.92	0.73	0.58–0.91
Physical work exposures				
Physical work exposures that pose problems				
Heavy physical exertion or lifting and carrying heavy loads	1.02	0.87–1.20	0.96	0.77–1.20
Uncomfortable working postures	1.21	1.04–1.41	1.28	1.03–1.58
Trunk rotation	1.04	0.89–1.20	0.79	0.63–1.00
Repetitive movements	1.20	1.03–1.39	1.18	0.96–1.45
Sitting	1.11	0.95–1.29	1.08	0.91–1.29
Standing	1.05	0.86–1.28	1.03	0.82–1.29
Behavior-related risk factors				
Smoking (ref: never)				
Past	1.13	0.95–1.35	1.06	0.86–1.29
Occasional	1.17	0.94–1.45	0.97	0.75–1.25
Current	1.30	1.05–1.61	1.19	0.91–1.55
Body mass index (ref: healthy weight)				
Underweight	0.81	0.47–1.41	0.61	0.31-1.19
Overweight	1.18	1.01–1.39	1.17	0.97–1.41
Obesity	1.24	1.01–1.52	1.17	0.93–1.47
Leisure-time physical activity (tertile, ref: low)				
Moderate	1.00	0.84–1.20	1.05	0.87–1.27
High	0.95	0.80–1.15	0.99	0.81–1.20
Binge drinking (ref: never)				
Less than once a month	1.02	0.85–1.22	0.96	0.79–1.17
Once a month	1.23	0.98–1.55	1.07	0.83–1.37
At least once a week	1.13	0.81–1.59	1.03	0.71–1.48

[1] Adjustment for age, education, place of birth, household income, father's education, mother's education, job strain, and for each other

[2] Age at first employment was not included in the final multivariable model, because it was not associated with self-rated health or common mental disorders after adjustment for two or more variables

Table S4: Age- and gender-adjusted odds ratios (OR) for the associations between workload factors and low back pain among participants with healthy weight, overweight or obesity and among participants with low level of leisure-time activity and in those with high level of leisure-time activity[1]

Workload factor	Healthy weight		Overweight or obesity		Low leisure-time activity		High leisure-time activity	
	OR	95% CI	OR	95% CI	OR	95% CI	OR	95% CI
Heavy physical exertion or lifting and carrying heavy loads	1.07	0.86-1.33	0.94	0.74-1.19	0.99	0.79-1.25	1.06	0.85-1.32
Uncomfortable working postures	1.25	1.02-1.53	1.12	0.89-1.41	1.16	0.94-1.44	1.27	1.02-1.58
Trunk rotation	1.10	0.89-1.35	0.92	0.73-1.15	1.00	0.81-1.24	1.08	0.87-1.34
Repetitive movements	1.38	1.12-1.69	0.99	0.79-1.25	1.17	0.95-1.45	1.25	1.01-1.56
Sitting	1.15	0.94-1.41	1.07	0.85-1.34	1.09	0.88-1.35	1.14	0.92-1.42
Standing	1.20	0.91-1.58	0.88	0.66-1.18	1.05	0.79-1.38	1.02	0.76-1.37
Vibration	1.40	0.84-2.33	0.75	0.45-1.25	1.11	0.67-1.86	0.96	0.58-1.58

[1] Median was used to dichotomize leisure time physical activity

Table S5. Age- and gender-adjusted odds ratios (OR) for the associations between age at first employment, workload factors and behavior-related risk factors

Characteristic	Smoking		Obesity		Physical activity	
	OR	95% CI	OR	95% CI	OR	95% CI
Age at first employment (ref: 22-24 years)						
≤18	3.00	2.34–3.85	1.67	1.32–2.11	1.03	0.85–1.24
19–21	2.04	1.62–2.58	1.84	1.50–2.26	1.01	0.86–1.18
≥25	0.77	0.58–1.02	0.77	0.61–0.98	1.07	0.91–1.27
Workload factors						
Physical exertion or lifting and carrying heavy loads (ref: no)						
Yes, they occur, but they pose no problems at all	1.63	1.28–2.07	1.55	1.27–1.91	1.29	1.10–1.50
Yes, they occur, and they do pose problems to a certain extent	2.23	1.80–2.77	1.51	1.24–1.84	1.29	1.12–1.50
Yes, they occur, and they pose major problems	2.38	1.72–3.28	1.52	1.11–2.09	1.23	0.97–1.57
Number of hours of physically demanding work per day (ref: 0)						
0.1–1	1.16	0.91–1.49	1.08	0.89–1.32	1.09	0.93–1.28
1.1–2.0	2.19	1.64–2.91	1.28	0.98–1.67	1.52	1.23–1.88
2.1–4.0	2.32	1.78–3.03	1.41	1.11–1.80	1.71	1.41–2.09
>4.0	3.08	2.42–3.91	1.40	1.11–1.75	1.67	1.39–2.00
Physical workloads that pose problems						
Heavy physical exertion or lifting and carrying heavy loads	1.89	1.58–2.26	1.30	1.10–1.54	1.18	1.04–1.34
Uncomfortable working postures	1.61	1.34–1.94	1.38	1.17–1.62	1.02	0.91–1.15
Trunk rotation	1.91	1.60–2.28	1.31	1.11–1.54	1.12	0.99–1.21
Repetitive movements	1.53	1.28–1.83	1.17	1.00–1.38	1.07	0.94–1.21
Sitting	0.66	0.55–0.80	0.95	0.81–1.12	0.84	0.74–0.95
Standing	1.47	1.18–1.83	1.45	1.19–1.77	0.90	0.77–1.07
Vibration	0.98	0.64–1.51	1.14	0.80–1.63	1.11	0.85–1.46

Table S6. Multivariable[1] odds ratios (OR) for the associations between gender, age at first employment, behavior-related risk factors, workload factors and behavior-related risk factors

Characteristic	Smoking		Obesity		Physical activity	
	OR[1]	95% CI	OR[1]	95% CI	OR[1]	95% CI
Gender, men vs. women	0.48	0.36–0.65	0.91	0.71–1.15	1.66	1.40–1.97
Age at first employment (Ref: 22-24 years)						
≤18	1.96	1.43–2.70	1.20	0.90–1.60	1.14	0.92–1.42
19–21	1.24	0.92–1.68	1.49	1.17–1.89	1.02	0.85–1.23
≥25	1.48	1.02–2.13	1.04	0.78–1.38	1.01	0.83–1.23
Behavior-related risk factors						
Smoking (ref: never)						
Past	-	-	1.25	1.01-1.56	0.92	0.77-1.09
Occasional	-	-	0.98	0.74-1.31	1.05	0.85-1.29
Current	-	-	1.17	0.89-1.55	0.71	0.56-0.91
Body mass index (ref: healthy weight)						
Underweight	1.99	1.00-3.94	-	-	0.94	0.59-1.49
Overweight	0.89	0.70-1.14	-	-	0.72	0.61-0.84
Obesity	1.09	0.83-1.44	-	-	0.45	0.36-0.55
Leisure-time physical activity (ref: low)						
Moderate	0.78	0.61-0.99	0.60	0.49-0.74	-	-
High	0.65	0.50-0.84	0.40	0.32-0.50	-	-
Binge drinking (ref: never)						
Less than once a month	3.05	2.17-4.27	1.20	0.96-1.49	0.92	0.78-1.07
Once a month	5.66	3.91-8.18	1.26	0.95-1.68	0.95	0.77-1.17
At least once a week	12.86	8.36-19.79	1.36	0.92-2.00	0.73	0.53-0.99
**Workload factors**						
Number of hours of physically demanding work per day (Ref: 0)						
0.1–1	1.10	0.81–1.50	1.14	0.90-1.44	1.19	1.00–1.41
1.1–2.0	1.66	1.14–2.41	1.05	0.76-1.46	2.07	1.62–2.64
2.1–4.0	1.64	1.13–2.38	1.08	0.78-1.50	2.35	1.84–3.02
>4.0	2.06	1.44–2.94	0.97	0.70-1.35	2.35	1.83–3.00
Uncomfortable working postures	1.02	0.76–1.37	1.27	1.00–1.61	0.88	0.74–1.06
Trunk rotation	0.97	0.72–1.31	0.93	0.72–1.21	1.04	0.86–1.27
Repetitive movements	0.94	0.72–1.23	0.84	0.67–1.05	1.02	0.86–1.22
Sitting	1.04	0.81–1.33	1.15	0.94–1.41	1.01	0.87–1.17
Standing	1.00	0.77–1.32	1.30	1.03–1.64	0.81	0.66–0.98
Vibration	0.55	0.33–0.91	0.94	0.63–1.40	1.02	0.75–1.39

[1] Adjustment for age, education, place of birth, household income, father's education, mother's education, job strain, and for each other
